# UV-Surface Treatment of Fungal Resistant Polyether Polyurethane Film-Induced Growth of Entomopathogenic Fungi

**DOI:** 10.3390/ijms18071536

**Published:** 2017-07-18

**Authors:** Gabriela Albara Lando, Letícia Marconatto, Felipe Kessler, William Lopes, Augusto Schrank, Marilene Henning Vainstein, Daniel Eduardo Weibel

**Affiliations:** 1 Laboratory of Photochemistry and Surfaces, Institute of Chemistry, Universidade Federal de Rio Grande do Sul (UFRGS), Av. Bento Gonçalves, 9500, CEP 91501-970 Porto Alegre, RS, Brazil; gaby.lando@gmail.com; 2 Laboratory of Geobiology, Institute of Petroleum and Natural Resources, Pontifical Catholic University Rio Grande do Sul (IPR–PUCRS), Av. Ipiranga, 6681, CEP 90619-900 Porto Alegre, RS, Brazil; leticiamarconatto@gmail.com; 3 Laboratory of Applied and Technological Physical Chemistry, Escola de Química e Alimentos, Universidade Federal do Rio Grande (FURG), Av. Itália, Km 08, CEP 96201-900 Rio Grande, RS, Brazil; felipekessler@gmail.com; 4 Laboratório de Fungos de Importância Médica e Biotecnológica, Departamento de Biologia Molecular e Biotecnologia, Centro de Biotecnologia, UFRGS, Av. Bento Gonçalves, 9500, CEP 91501-970 Porto Alegre, RS, Brazil; lopeswlm@gmail.com (W.L.); aschrank@cbiot.ufrgs.br (A.S.); mhv@cbiot.ufrgs.br (M.H.V.)

**Keywords:** polyether polyurethane, *Metarhiziumanisopliae*, UV, abiotic treatment, acrylic acid

## Abstract

Synthetic polymers are the cause of some major environmental impacts due to their low degradation rates. Polyurethanes (PU) are widely used synthetic polymers, and their growing use in industry has produced an increase in plastic waste. A commercial polyether-based thermoplastic PU with hydrolytic stability and fungus resistance was only attacked by an entomopathogenic fungus, *Metarhiziumanisopliae*, when the films were pre-treated with Ultraviolet (UV) irradiation in the presence of reactive atmospheres. Water contact angle, Fourier transform infrared spectroscopy in attenuated total reflection mode (FTIR-ATR), scanning electron microscopy (SEM), and profilometer measurements were mainly used for analysis. Permanent hydrophilic PU films were produced by the UV-assisted treatments. Pristine polyether PU films incubated for 10, 30, and 60 days did not show any indication of fungal growth. On the contrary, when using oxygen in the UV pre-treatment a layer of fungi spores covered the sample, indicating a great adherence of the microorganisms to the polymer. However, if acrylic acid vapors were used during the UV pre-treatment, a visible attack by the entomopathogenic fungi was observed. SEM and FTIR-ATR data showed clear evidence of fungal development: growth and ramifications of hyphae on the polymer surface with the increase in UV pre-treatment time and fungus incubation time. The results indicated that the simple UV surface activation process has proven to be a promising alternative for polyether PU waste management.

## 1. Introduction

Polyurethanes (PUs) are very common synthetic polymers present in modern life with a wide range of applications. Over the past few decades, PUs have been replacing other polymers in many industrial and service sectors, such as latex rubber in car interiors and seats because of their lower density and flexibility [1]. PUs are also used as coatings because they exhibit excellent adhesion to diverse types of surfaces, adding abrasion resistance and electrical isolation to the material. PUs also have additional advantages in durability. Due to its high melting point and high value of tensile strength, when compared to other polymers, it has shown great resistance to degradation by solvents, oils, and water [1]. Paradoxically, the wide range of applications and uses of plastics, in general, and PUs, in particular, is the reason synthetic polymers cause some major environmental impacts. A large proportion of plastic waste is directed to landfill sites where their high durability and their low degradation rates increase pollution problems in water and on land. Recycling is yet to provide a safe solution for the disposal of all plastic waste. In Brazil, for example, in spite of the ABNT NBR 13,230 Brazilian standard, which was issued more than 20 years ago, less than 20% of both rigid and film plastic is recycled on average. The main problems are misinformation and the incorrect resin identification codes being used [2].

According to recent publications, alternative waste management strategies have to be developed to resolve water and land pollution problems [3,4,5,6,7,8]. In this sense, biodegradation is a green option to solve pollution issues. The biodegradation process is catalyzed by the extracellular enzymes that are secreted by microorganisms. A recent review shows that several microbial enzymes, able to modify or degrade synthetic polymers, have been identified in recentyears [9]. By those biocatalysis mechanisms the microorganisms, such as bacteria and fungi, can start to assimilate the polymer fragments and mineralize them completely in CO_2_, methane, water, and biomass [10]. Biodegradation of synthetic polymers is usually a heterogeneous process where the high molecular size of the polymer, its water insolubility, and the surface hydrophobicity avoid the incorporation of the carbon source directly into the cells, where they can be metabolized [10].

Previous studies have shown that fungi are the dominant microorganisms involved in the biodegradation of polyester PU when buried in soil [11,12]. Several works have identified various fungal communities that are active in the colonization of PUs [8,12,13]. In a recent review that focused on the actual enzymes that attack PUs, it was shown that, in spite of the promising results of the research carried out, there is a lot of information in relation to bacteria and fungi that are capable of degrading PUs still to be confirmed [6]. The authors pointed out that the research carried out to understand the enzymatic mechanisms of biodegradation on PUs are for polyester Pus, and not for polyether PUs, the latter being the most difficult to biodegrade. A very recent work has shown that polyester hydrolases from actinomycetes are able to degrade polyester PU materials [14]. Several polyester hydrolases confirmed important degradation activities in dispersed and solid polyester PUs. By a turbidimetric assay the authors were able to analyze the fast kinetics of enzymatic PU hydrolysis proving the important role of enzymes for the biodegradaton of synthetic polymers.

There are also reports showing that abiotic factors, such as photodegradation or hydrolysis, play a minor role in the degradation of plastics and that the microorganisms are responsible for the majority of plastic degradation [13,15]. Our recent work has shown that simple surface modification processes, such as the use of ultraviolet (UV) light in the presence of reactive atmospheres can efficiently activate biomechanisms [16,17,18]. For example, controlled degradation of poly(3-hydroxy-butyrate), PHB, films by entomopathogenic fungi was achieved using a UV-assisted surface modification of PHB in the presence of oxygen [17]. In another example, the surfaces of the poly(sulfone) and PU films were modified by UV-assisted treatments to improve their wettability, adhesion, and cell spreading properties. Treated films showed comparable results in the number of adhered cells to the control group. Untreated films showed a lower number of adhered cells when compared to the treated films, showing that cell adhesion was influenced by the treatments, which increased the biocompatibility [16]. The synergetic effect of the abiotic and biotic process in the biodegradation of synthetic polymers was also studied and reviewed recently [19,20,21,22,23].

In this study, a commercial polyether-based thermoplastic polyurethane that exhibits abrasion resistance, hydrolytic stability, and fungus resistance was chosen. The surface properties of PU films were permanently modified to hydrophilic conditions in different degrees by UV-assisted treatment in the presence of oxygen gas or acrylic acid (AA) vapors. The presence of poly(acrylic acid) (PAA) can be toxic to some types of cells, such as muscle [24] and corneal epithelial cells [25]. However PAA films have also shown positive effects, for example, the biodegradation of superabsorbent polymers in soils when linear PAA was used to form a composite [26], in the adsorption of proteins and peptides [27], and the increase in biodegradation of composites containing grafted-AA [28]. The AA treatments appear to be a way to introduce carboxylic groups on the polymer surface which allow the immobilization of molecules with amino groups in their structures [29,30].

The effect of the UV-assisted treatments to activate the growth of the entomopathogenic fungus, *Metarhiziumanisopliae*, was studied. A polyether PU with resistance to fungus and hydrolytic attack has been studied. It only lost its original properties in the presence of *Metarhiziumanisopliae* after the PU films were treated with UV in the presence of reactive atmospheres. On the contrary, the pristine polyether PU appeared largely unaffected in the presence of fungus if no surface modification treatment was used. The results indicate that the simple UV surface activation process is a potential alternative for polyether PUs waste management.

## 2. Results

### 2.1. Surface Characterization of the Polyether Polyurethane Films

**Figure 1:**
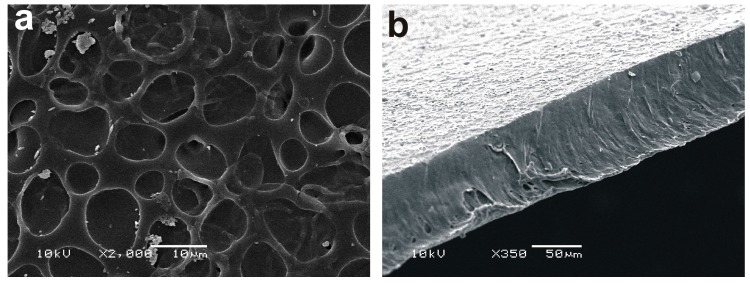
Images of a typical pristine Polyurethane film prepared by casting: (**a**) top scanning electron microscopy (SEM) image and (**b**) SEM cross-section image.

PU films with an average thickness of about 70 μm were prepared using the casting technique. SEM top and cross-section images of a typical pristine film can be seen in Figure 1a,b, respectively. The roughness, measured using profilometry, of the film was below 50 nm (root mean squared roughness, RMS).

Water contact angle (WCA) measurements of pristine and UV-treated PU films were carried out. The results showed that PU films are slightly hydrophobic showing a WCA of about 92° (see Table S1).

**Figure 2:**
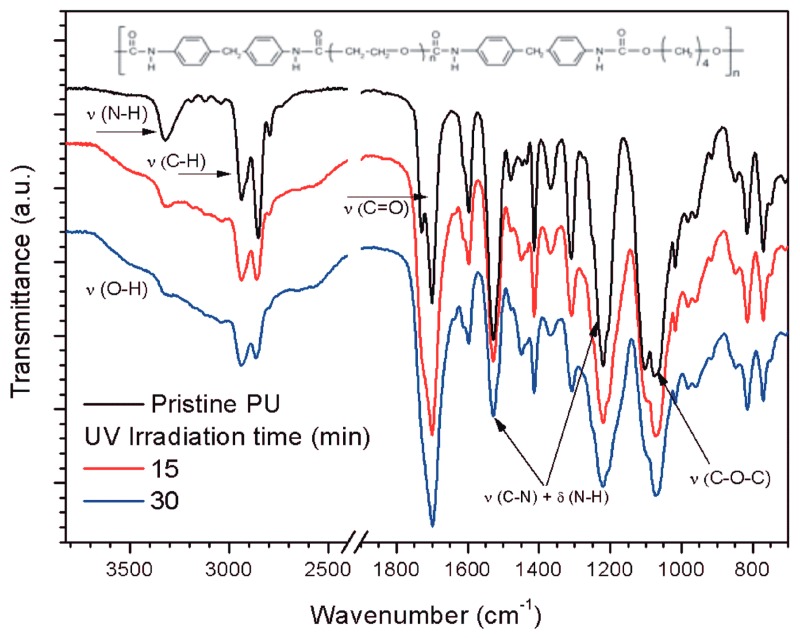
Fourier transform infrared spectroscopy in attenuated total reflection mode (FTIR-ATR) spectra of pristine PU films and treated ones with UV irradiation in the presence of acrylic acid (AA) vapors for different times. The spectra were slightly moved in the vertical direction for better presentation. The molecular structure of the polyurethane studied here is shown at the top of the figure.

The FTIR-ATR spectrum of pristine PU (Figure 2) shows the main bands, characteristic of the polymer: N-H stretching (3325 cm^−1^), C-H asymmetric and symmetric stretching (2940 cm^−1^ and 2855 cm^−1^, respectively), C=O stretching (1730 and 1701 cm^−1^), and C-O stretching (1218 and 1076 cm^−1^) [31,32]. The molecular structure of the PU used here is shown in the inset in Figure 2.

### 2.2. Surface Modification of the Polyether PU Films by Ultraviolet-Assited Photochemistry

When PU films were irradiated in the presence of oxygen or AA vapors a decrease in the WCA with an increase in the irradiation time was observed for both treatments (see Table S1). The data agrees with our previous work on the surface functionalization of PU films obtained by a UV-assisted modification in the presence of oxygen or AA vapors using a different experimental setup [33].

FTIR-ATR analyses of the PU films treated with UV in the presence of oxygen or AA vapors showed important differences in the surface modification processes. UV treatment in the presence of oxygen mainly indicated the incorporation of OH groups in the surface region (see Figure S1). The N–H signal appeared almost unmodified in relative intensity when the irradiation time increased. Carbonyl signal intensities increased slightly when the irradiation time increased (see Figure S1). The results with oxygen contrasted with the results of the treatments using AA vapor during UV irradiation. The wide signal of the OH functional groups is present; however, new features merged in the polymer spectrum (see Figure 2). N–H and C–H signals almost disappeared after only 30 min of irradiation. This is the opposite result from the oxygen treatments, in which the carbonyl stretching signal increased relative to N–H. An increase in the C–H stretching signal intensity was observed in relation to the increase in photolysis time.

**Figure 3:**
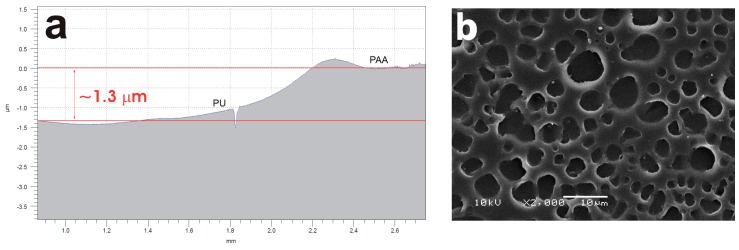
Profilometer cross-section of a PU film after UV irradiation in the presence of AA vapors for 30 min (**a**); SEM image of PU film irradiated with UV in the presence of AA vapors for 30 min (**b**).

UV-assisted treatments of polymers usually preserve the original roughness of the polymeric substrates which contrasts with other surface modification techniques, such as plasma treatments [18,34]. Contact profilometry measurements were carried after UV irradiation of the PU films in the presence of oxygen and AA vapors. For example, it was observed that a film of about 1.3 μm was formed on top of the PU film after 30 min of UV treatment when AA vapors were used (see Figure 3a). The roughness of the measured film was about 80 nm. Previous reports have shown that AA plasma treatments [29,35,36,37,38,39,40] or UV photolysis of AA vapors leads to a polymerization process of the monomers forming a poly(acrylic acid) (PAA) like film on top of the films [18,33]. The PAA film formed due to the UV polymerization process, keepingthe porous structure of the surface (see Figure 3b) when compared with the UV-untreated film (see Figure 1a). Apart from this, the difference spectra “pristine PU–PU treated with AA vapors for 15 or 30 min” (see Figure S3) showed the typical pronounced features of a PAA film [41]. The intensity of the PAA characteristic signals increased with the increase in irradiation time.

### 2.3. Induced Growth of Entomopathogenic Fungi

**Figure 4:**
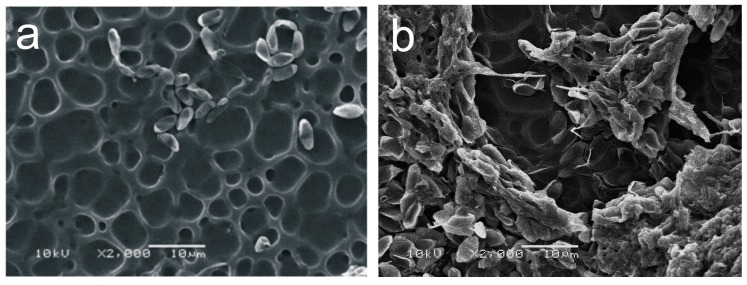
SEM Images of PU films incubated for 60 days in the presence of entomopathogenic fungus *Metarhiziumanisopliae*: (**a**) untreated film; and (**b**) UV treated film in an oxygen atmosphere for 120 min.

SEM images of PU without treatment and incubated for 10, 30, and 60 days did not show any indication of fungal growth (see Figure 4a for an incubation time of 60 days). In Figure 4a no hyphae, that would indicate fungal growth, was observed. Only the inoculated spores are observed.

When PU films were irradiated with UV (30 min) in the presence of oxygen, inoculated with *M. anisopliae* and incubated for 30 days, it was possible to observe the presence of a few hyphae, indicating low fungal growth rates and surface fixation (see Figure S3). The development and penetration of a few hyphae after the PU film was irradiated with UV light for 120 min in the presence of oxygen and incubated for 60 days with the fungus is observed in Figure 4b. After incubation, the films were extensively washed with distilled water under ultrasonic treatment. Figure 4b shows a layer of fungi spores all over the sample, which indicates great adherence of the microorganisms to the polymer.

**Figure 5:**
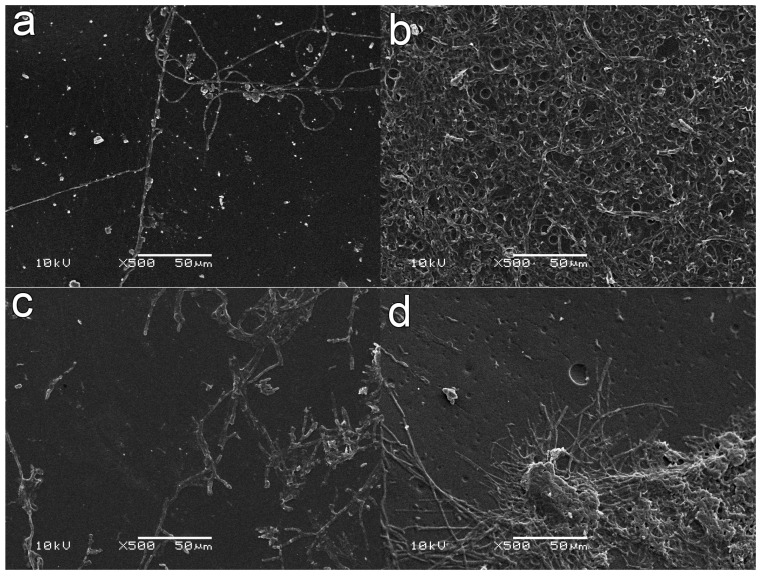
SEM images of PU cultivated in the presence of entomopathogenic fungus *Metarhiziumanisopliae* for 10 (**a**,**c**) and 30 days (**b**,**d**). The films were irradiated with AA vapors for 15 min (**a**,**b**) and 30 min (**c**,**d**) before incubation.

SEM images of samples treated with AA for 15 min and after 10 days of cultivation showed the clear presence of adhered hyphae on the polymer (Figure 5a). These hyphae growths were not clearly observed on the UV-oxygen treated films (See Figure 4b and Figure S3). After 30 days of incubation (Figure 5b), it was possible to observe an evident increase in fungal development.

PU films irradiated with UV in the presence of AA vapors for 30 min and incubated for 10 days (Figure 5c) seem to show a higher rate of hyphae development and ramification compared with samples treated with AA for 15 min and incubated for the same period (Figure 5a). A similar result was observed when the incubation time was 30 days, showing an increased amount of fungal hyphae development and ramifications even after ultrasonic washing (Figure 5d).

**Figure 6:**
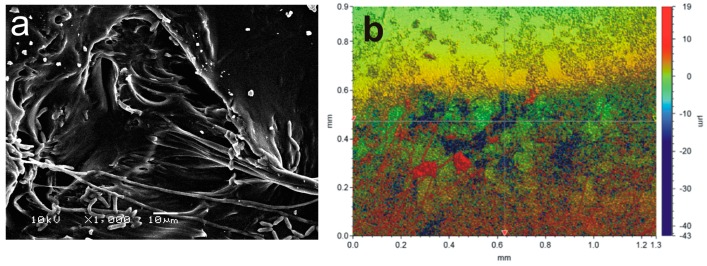
SEM image of PU films irradiated with AA vapors for 30 min and incubated in the presence of the entomopathogenic fungus *Metarhiziumanisopliae* for 30 days (**a**). Profilometer results of the same PU films (**b**).

More detailed SEM and optical profilometer data were obtained for PU films, which were UV-irradiated with AA for 30 min and incubated for 30 days. The presence of a high concentration of fungal hyphae development and ramification was observed even after extensive ultrasonic washing (see Figure 6a). Additionally, optical profilometer measurements showed that the roughness of the surface strongly increased reaching an RMS value of (0.4 ± 0.1) µm (see Figure 6b). The profilometer cross-section of these PU films (see Figure S4) clearly shows that the surface roughness increased compared to the films before incubation (see Figure 3). These results unequivocally showed that the surface activation process has a dramatic effect on the fungal hyphae development and ramification.

**Figure 7:**
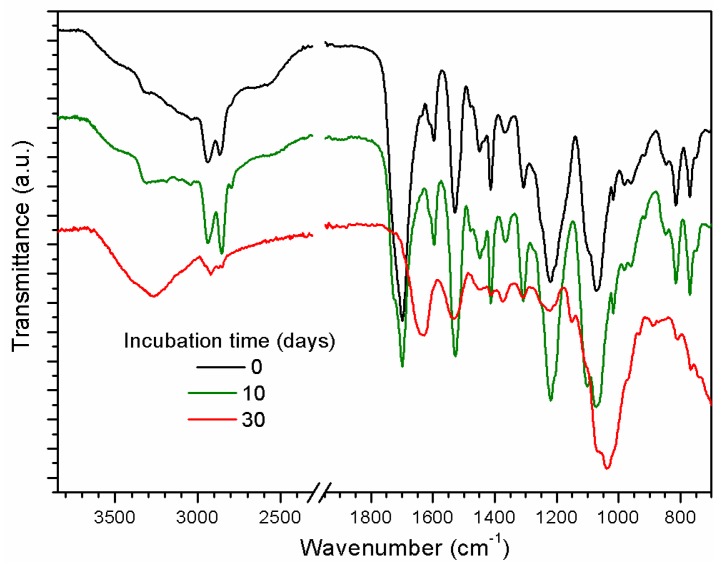
FTIR-ATR spectra of PU films treated with UV in the presence of AA vapors for 30 min without incubation and after incubation for 10 and 30 days. The spectra were slightly moved in the vertical direction for better presentation. For the assignments of the main bands refer to Figure 2.

**Figure 8:**
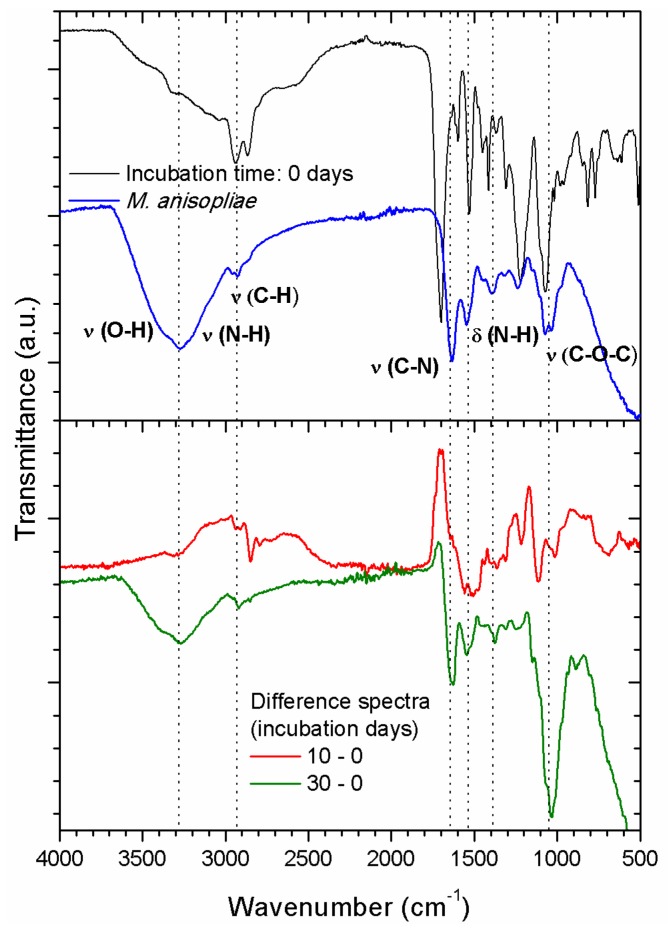
FTIR-ATR spectra of PU films treated with UV in the presence of AA vapors for 30 min without incubation (**top**) along with their difference spectra (**bottom**) and after incubation for 10 and 30 days. The FTIR-ATR spectrum of the fungus without any polymer is shown also on top together with the main signal assignments. The spectra were slightly moved in the vertical direction for better presentation.

FTIR-ATR spectra of PU films, which were UV-irradiated with AA for 30 min and incubated for 10 and 30 days are presented in Figure 7. It is possible to observe a slight increase in the O-H and broadening of N-H (3600–3000 cm^−1^) bands. More interesting, all the other relevant bands of the PAA and even PU are reduced according to the increase in incubation time, indicating the build-up of a thick layer on top of the PU/PAA films. This thick fungus layer reduced the characteristic FTIR-ATR signals of the PU substrate, adding evidence to the profilometer measurements about the presence of a thick fungus layer on top of the original PU film. There is a clear similarity between the FTIR-ATR spectrum of the fungus without any polymer that it is shown in Figure 8 (top) and the spectrum obtained for 30 days of incubation (see Section 2.4).

### 2.4. Weight Change Measurements

**Figure 9:**
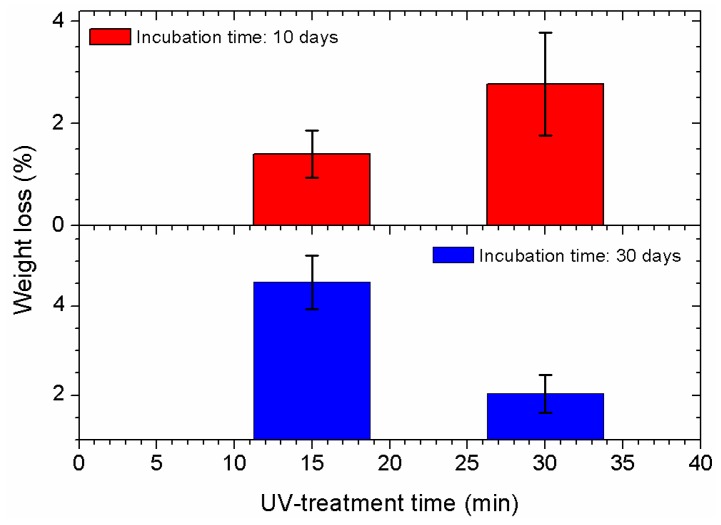
Weight loss results for PU films untreated and treated with UV in the presence of AA vapors for 15 or 30 min. Incubation time: 10 (**red**) and 30 (**blue**) days.

Figure 9 shows the weight loss results for the PU films with UV-assisted treatment in the presence of AA vapors after two periods of incubation with *M. anisopliae*. Untreated samples did not show any weight loss. PU films exposed to UV in the presence of oxygen and incubated for 15 and 30 days did not reveal significant weight loss [42]. When PU films were irradiated with UV light for 120 min in the presence of oxygen and incubated for 60 days with the fungus, an increase in weight was measured. In this case, a layer of fungi spores all over the sample was observed, indicating great adherence of the microorganisms to the polymer (see Figure 4b). A weight decrease with incubation time can be observed when AA vapors were used during the UV treatments (see Figure 9) indicating an average weight loss of about 3%. When the incubation time was 30 days, the weight loss peaked at about 4.5% (15 min of UV irradiation) and then decreased to about 2% when the UV irradiation time was 30 min. This last result may indicate larger amounts of fungus ramifications and hyphae presented in the PU films that could not be removed by washing with ultrasound assistance (see Figure 6).

The evidence of the presence of larger amounts of fungus ramifications and hyphae can be seen when the FTIR-ATR spectra of Figure 6 are compared in more detail. Figure 8 (bottom) shows the difference spectra between a PU film treated with UV in the presence of AA vapors for 30 min without incubation (Figure 8 (top)) and PU films with the same treatment but after incubation for 10 and 30 days. The subtraction was carried out minimizing the main bands, characteristic of the polymer: N-H stretching (3325 cm^−1^), C-H asymmetric and symmetric stretching (2940 cm^−1^ and 2855 cm^−1^, respectively). As can be seen in Figure 8 (bottom) when the film was incubated for 30 days the FTIR-ATR difference spectrum resembles the spectrum of the entomopathogenic fungus *Metarhiziumanisoplia*e. The typical carbonyl PAA signal almost also disappeared. On the contrary when the PU film was incubated for 10 days the fungus signals are much weaker and the carbonyl signal of the PAA is evident (compare with Figure S2). These results corroborate the presence of a thick fungus layer when the incubation time was 30 days. Similar data treatments were carried out for PU films treated with UV in the presence of AA vapors for 15 min and PU films with the same treatment after incubation for 10 and 30 days. The obtained results were less conclusive. Figure S5 showed the presence of thinner fungus layers because, in both incubation times, the carbonyl signal of PAA was evident.

## 3. Discussion

In the present work, the ability of *M. anisopliae* to attack the fungus-resistant polyether PU (1185A10) films was investigated. An important fungal activity was only found when the films were pre-treated with UV irradiation in the presence of reactive atmospheres (oxygen or AA vapors). Only samples that were previously UV treated showed fungal hyphae development and ramification. Longer irradiation times and incubation periods revealed a higher rate of hyphae development and ramification. These results were particularly evident when AA vapors were used during the UV treatments.

Pristine films showed a hydrophobic surface with a WCA of 92° (see Table S1), which means that it is a non-favorable surface for water-soluble enzyme molecules. In previous studies, it was shown that insoluble PU substrates can be degraded by enzyme molecules which, by an unknown process, attach themselves onto the surface of the insoluble substrates [43]. After the adhesion process, the enzymatic reaction may rapidly proceed in some cases [1]. However, the degradation by enzymes are common for polyester PUs [1,6,14,44,45], but not for polyether PUs. The rate of polyester PU degradation by enzymes, bacteria, and fungus are usually much higher than that for polyether PUs. In a recent review, several examples of the low degradation rate of polyether PUs are presented [6]. Kay et al., for example, have suggested that the urethane link in the monomer unit of the polymer is the main site where the attack starts [46]. In the case of polyester PUs, the polyester chain seems to be the site of attack during the first stages of biodegradation. It was also pointed out that the main difficulty in understanding the mechanisms of polyether PU biodegradation is due to the low degradation rates of these polyether PUs, which limits the research work [6]. The results presented in Figure 4a agree with the previous works. Untreated PU films incubated in the presence of *M. anisopliae* for 10, 30, and 60 days did not show any hyphae growth, which would indicate fungal development. Only the inoculated spores were observed (see Figure 4a).

The situation changes drastically when the surface activation process by UV irradiation in the presence of reactive atmospheres is used as a pretreatment method of the PU films before fungus incubation. Abiotic pre-treatments, such as thermal or UV radiation are known to have important synergistic effects in the biodegradation of polymers [21,22,47]. In particular, in a recent study, a detailed investigation of the effect of UV irradiation on polyurethanes was carried out [48]. Rosu et al. found evidence that the absorption of UV light modified the chemical structure of PU, inducing degradation and photooxidation of the CH_2_ groups. Those photochemical reactions give rise to the typical yellowing of the PU surfaces. When PU films were irradiated in the presence of oxygen for 30 min with UV light and then cultivated for 30 days, it was possible to observe a minimal presence of hyphae and a layer of fungi spores all over the sample, indicating great adherence of the microorganisms to the polymer (see Figure 4b and Figure S3b). Similar results were obtained when PU films were treated with UV light for 120 min in the presence of oxygen and cultivated for 60 days (see Figure 4b and Figure S3c).

There are several studies showing the synergistic effects of an abiotic and biotic process during biodegradation of different polymers. In this sense, the type and number of functional groups in the surface region is an important component of the biodegradation process. In addition, the change in concentration of several groups can be used as sensors that indicate the presence of biotic degradation [19,39,40]. In general, it is accepted that the surface wettability is an important property of the surface in the starting stages of the biodegradation of a polymeric material which will probably determine the extent of the substrate colonization with microorganisms [22,49]. The results presented with the UV pre-treatment of the PU films in the presence of oxygen showed the importance of the surface hydrophilicity in the surface activation process (see Table S1, Figures S1 and S3). The presence of carboxylic functional groups on the surface represents new possibilities for biotic processes [5,22,50,51]. Additionally, carboxylic, esters, and vinyl functional groups together with the presence of double bonds are the main indicators of changes that show the activity of microorganisms on substrates surfaces [21]. For example, the reduction of carboxyl groups and the corresponding increase in double bonds in the presence of microorganisms is an indication of biotic activity.

PAA films are an important source of carboxyl groups, but the presence of PAA can be toxic for some types of cells. For example, human smooth muscle cell growth was carried out on pristine poly(ethylene terephthalate) (PET) films and on a modified PET surface with PAA [24]. In PAA-modified PET surfaces the cells did not show the tendency to adhere to the surface and died quickly. Similar results were also obtained for corneal epithelial cells when PAA was grafted onto silicon membranes [25]. PAA films, however, have also shown positive effects, for example in the same work carried out on PET films [24], the authors found that the PAA-grafted PET surface with collagen, shows excellent adherence and rapid cell growth. The rate of cell growth was high enough to cover the complete film surface with cells within a period of six days. In a recent study about the biodegradation of synthetic superabsorbent polymers to increase the water availability for plant growth in soils, it was shown that the inclusion of linear PAA to the cross-linked PAA increased the overall biodegradability of a PAA-based superabsorbent [26]. Synchrotron-based soft X-ray photoemission electron microscopy (X-PEEM) studies of positively-charged peptides and negatively-charged protein interaction with biomaterial surfaces showed that the maximum absorptions were observed when a layer of PAA was present [27]. In an investigation into the biodegradation of agricultural composites residues containing grafted-AA acrylic, a strong correlation between biodegradation and the concentration of AA in the composite was found [28]. The correlation suggests that AA treatments are a way to introduce carboxylic groups on the polymer surface, which allows the immobilization of molecules with, for example, amino groups in their structures [29,30].

PU films pre-treated with UV light in the presence of AA vapors showed the presence of a thin PAA layer on top of the PU film (see Figure 3a). The photo-polymerization process led to an increase of carboxyl functional groups on the surface region when the photolysis time increased (see Figure 2 and Figure S2) [16,23,32]. The same result is observed for the OH signal. SEM images of PU films treated with UV light in the presence of AA vapors for different periods of time and incubated for different time periods clearly showed the presence of adhered hyphae on the polymer (Figure 5 and Figure 6). After 30 days of incubation (Figure 5b), it is possible to observe an even higher increase in the fungal development and ramifications compared to 10 days of incubation, which may indicate a more favorable surface environment for fungus growth. FTIR-ATR results also corroborate the SEM observations. Carboxylic functional groups decreased when the incubation time increased, disappearing completely for the films incubated for 30 days. Figure 7 also shows that the spectra obtained for 30 days of incubation in the presence of *M. anisopliae* present new intense FTIR-ATR signals. Alkenyl C=C stretching signals (1680–1620 cm^−1^) overlaps the 1560–1650 cm^−1^ signals, which are related to the N–H stretching and N–H bending of urethane group. The typical N–H signal stretching at about 3330 cm^−1^ of the PU that almost disappeared with the increase in UV irradiation time in the presence of AA vapors (see Figure 2). In Figure 7 the N–H signal stretching is wider and the intensity apparently increases with the increase of the incubation time. Figure 8 (top) shows a typical FTIR-ATR spectrum of *M. anisopliae* without polymer. The similarity with the FTIR-ATR spectrum of the PU film treated with UV in the presence of AA vapors after incubation for 30 days is evident. These results show the strong activity of the fungus on the surface of the PU films when a layer of PAA is present.

Finally, a few measurements were carried out trying to detect weight losses after the treatments. The weight loss test is usually applied in degradations experiments of films and substrates, although it is not a direct proof of biodegradation due to several problems, such as sample disintegration, precautions in sample preparations, etc. [52]. Nevertheless, the results shown in Figure 8 revealed the activation of a surface process when UV treatments in the presence of AA vapors were used. After the incubation of these PU-treated substrates, plenty of fungus ramifications and hyphae were present on the PU film’s surface that could not be removed by washing with ultrasound assistance. The weight loss measurement was about 3% on average. Weight losses in biodegradation studies of polymers have been widely measured trying to add new data that can support the results [8,14,53,54,55,56]. For example, in a recent study of enzymatic degradation of solid thermoplastic polyester PU (TPU) weight losses of up to 4.9% and 4.1%, depending on the type of TPU used, were determined at 70 °C and within a reaction time of 200 h [14]. In other work, three series of TPUs were synthesized and the effect of chain extender structure on the degradation was investigated [57]. In some of the series studied, the authors found weight losses between 0% and 100% after 365 days of incubation. In other series the weight loss was lower than 7% after 365 days of incubation. The author’s conclusion was that important weight losses were observed for those TPUs only when the molecular weight decreased below 6000 mass units during the degradation. In an interesting study of the effect of an abiotic process (UV irradiation) on the biodegradation of poly(3-hydroxybutyrate) (PHB) the authors found evidence that the initial delay in the biodegradation was due to the presence of a thin superficial layer of high crystallinity. After this layer is consumed the biodegradation rate increased, but the final weight losses measured after more than 300 days of incubation were below 6.5% [56]. The above studies have related some of the reasons that can retard the initial stages of a biodegradation process, even in a biodegradable polymer. The particular polyether PU studied here exhibits abrasion resistance, hydrolytic stability, and fungus resistance. The measured weight loss of about 3% in only 10–30 days of incubation happens only when the films were previously treated with UV in the presence of AA vapors. These results show the important effect of the abiotic processes in the surface activation of synthetic polymers.

The results presented here confirmed the important effect on the activation of the *M. anisopliae* growth mechanisms only when the films were pre-treated by UV irradiation in the presence of reactive atmospheres, in particular when a PAA film was formed on top of the PU.

## 4. Materials and Methods

### 4.1. Materials

PolyurethaneEllastolan^®^ (PU 1185A10) was purchased from BASF^®^(Ludwigshafen, Germany). Tetrahydrofuran (THF) and acrylic acid >99% were obtained from Sigma Aldrich (São Paulo, Brazil). Oxygen (99.99%) and nitrogen (99.999%) were purchased from Air Liquide Brazil Ltda (Canoas-RS, Brazil). All materials were used as received.

### 4.2. Film Preparation and UV-Surface Modification

PU films were prepared using the casting technique from a 5 × 10^−4^ mol.L^−1^ THF solution. Surface modification was carried out using UV irradiation at room temperature following the procedure previously described [16,17,18,33]. Briefly, a controlled flow of oxygen gas was introduced into a homemade bench reactor containing five low-pressure mercury lamps (6W, Orion, Bristol, UK). AA vapors were introduced into the reactor using nitrogen as the gas carrier (2.5 cm^3^·s^−1^). PU samples, 2 cm × 2 cm in size, were irradiated at constant intervals (0, 15, and 30 min). All samples were washed with 500 mL of deionized water after the irradiation and dried in a desiccator.

### 4.3. Surface Characterization

Water contact angles (WCA) of unmodified and UV-modified PU films were measured at room temperature after 24 h of treatment. The wettability measurement was performed using the sessile drop method using a Krüss DSA30 (Krüss GmbH, Hamburg, Germany). The data acquisition and processing were carried out using the “Drop Shape Analysis System” software (Version 2.1, Krüss GmbH, Hamburg, Germany) and the values reported are the averages of three to six measurements performed in different areas on each sample surface.

Fourier transformed infrared spectroscopy measurements (FTIR) were performed using an Alpha-P model, Bruker (GmbH, Hamburg, Germany), with a spectral resolution of 4 cm^−1^. Attenuated total reflectance (ATR) module with diamond prism was used at an incidence angle of 45°.

Scanning electron microscopy analyses (SEM) were performed using an EVO50-Carl Zeiss electron microscope at 10 kV. The average thickness of the films was measured using SEM images (JSM 6060, Jeol Ltd., Peabody, MA, USA). Ultrasonic washing was carried out before SEM measurements if the films were incubated in the presence of *Metarhiziumanisopliae*.

Surface roughness measurements were carried out using an Ambios XP-2 profilometer and an optical profilometer, Contour GTK 3D optical profiler. In optical profilometry, the data analyses and the root-mean-square roughness (RMS) were calculated using the software “Vision64” (Version 5.41 update 4, Bruker, Tucson, AZ, USA).

### 4.4. Weight Change Measurements

Pristine PU films were dried in a desiccator at room temperature for several days and weighted (W_0_). After incubation the films were washed with ultrasound assistance in distilled water for 15 min and dried in a desiccator at room temperature also for several days. Finally they were weighted several times until the same weight during three days of weighting (W_S_)was obtained. Weight changes (%) of the PU samples were calculated using Equation (1):

Weight change (%) = ((W_0_ − W_S_)/W_0_) × 100% (1)

### 4.5. Fungi Maintenance

The entomopathogenic fungus *M. anisopliae* CG97 was obtained from the Laboratory of Cellular and Molecular Biology of Filamentous Fungi collection, from Universidade Federal de Rio Grande do Sul. The fungus was kept in Cove complete medium [58] at 4 °C, following the procedure previously described [17].

### 4.6. Fungi Inoculationand Growth Behavior Evaluation

Three samples of pristine and modified PU films were washed with 70% ethanol solution for sterilization and placed in Petri dishes containing a minimal nutrient medium (MNM) to evaluate growth behavior. Taking into account that other sterilization methods such as autoclaving (steam sterilization) or ultraviolet light would affect the polymer surface, a 70% ethanol solution was used, which was efficient as there was no growth of any microorganism in the control experiment. Additionally, 70% ethanol was also highly efficient in stopping further fungus development. The MNM (*w*/*v*) contained: glucose 1%, NaNO_3_ 0.6%, and agar 1.5%. A salt solution containing (*w*/*v*): KCl 2.6%, Mg_2_SO_4_.7H_2_O 2.6%, and KH_2_PO_4_ 7.6%, also trace elements: Na_2_B_4_O_7_. 7H_2_O 0.004%, CuSO_4_. 5H_2_O 0.04%, FeSO_4_ 0.001%, MnSO_4_. 2H_2_O 0.08%, Na_2_Mo_4_. 2H_2_O 0.08% and, ZnSO_4_. 7H_2_O 0.08% was added to the medium, which was 2% of the total volume. Approximately 10^7^ fungus spores were inoculated at the center of polymeric samples and incubated at 28 °C for 10 and 30 days. After the incubation time, the samples were washed again in a 70% ethanol solution and submerged in deionized water and washed with ultrasound assistance in order to eliminate fungi present on the polymeric surface. Non-inoculated Petri dishes acted as the control. The amount of medium added to each plate was 32 mL. The culture volume compared to the amount of PU films used in each Petri dish was not calculated. The whole experiment was carried out in a solid medium. All measurements were made in triplicate.

## 5. Conclusions

In this study, it was demonstrated that commercial polyether-based thermoplastic PU films that exhibit abrasion resistance, hydrolytic stability, and fungus resistance can be attacked by entomopathogenic fungi only if the films have been pre-treated with an abiotic process: UV irradiation in the presence of reactive atmospheres. Due to the UV-assisted pre-treatments in the presence of oxygen gas or acrylic acid vapors, the surface properties of the PU films were permanently modified to be hydrophilic with different types and amounts of functional groups. The effect of these treatments on the degradation of PU films by *M. anisopliae* was investigated. Oxygen pre-treatment showed a layer of fungi spores all over the sample indicating great adherence of the microorganisms to the polymer. However, the UV treatment in the presence of AA vapors led to a remarkable growth of the entomopathogenic fungi. SEM and FTIR-ATR data showed clear evidence of the adhered hyphae on the polymer. The fungal development and ramifications increased with the pre-treatment time and incubation time. Additionally, FTIR-ATR data showed that the carboxylic signals from PAA disappeared with the increase of incubation time and the appearance of new signals in corresponding amounts, which were similar to pure *M. anisopliae*. On the contrary, the pristine polyether PU appeared largely unaffected in the presence of fungus if no surface modification treatment was used. The results indicate that the simple UV surface activation process has proven to be a promising alternative for polyether PU waste management.

## Supplementary Materials

The following are available online at www.mdpi.com/1422-0067/18/7/1536/s1.
