# Supplementary material for: UV-Surface Treatment of Fungal Resistant Polyether Polyurethane Film-Induced Growth of Entomopathogenic Fungi
Source: Int J Mol Sci. 2017 Jul 18;18(7):1536. doi: 10.3390/ijms18071536 (PMC5536024; doi:10.3390/ijms18071536)
Supplement: Supplementary file 1 [file ijms-18-01536-s001.pdf]

Supplementary Materials

UV-Surface Treatment of Fungal Resistant Polyether Polyurethane Films Induced Growth of Entomopathogenic Fungi

Gabriela Albara Lando[1,], Letícia Marconatto[2], Felipe Kessler[3], William Lopes[4], Augusto Schrank[4], Marilene Henning Vainstein[4] and Daniel Eduardo Weibel[1,*]

[1] Laboratory of Photochemistry and Surfaces, Institute of Chemistry – Universidade Federal de Rio Grande do Sul (UFRGS), Av. Bento Gonçalves, 9500, CEP 91501-970, Porto Alegre - RS – Brazil
[2] Laboratory of Geobiology, Institute of Petroleum and Natural Resources, Pontifical Catholic University Rio Grande do Sul (IPR – PUCRS), Av. Ipiranga, 6681, CEP 90619-900 – RS – Brazil
[3] Laboratory of Applied and Technological Physical Chemistry, Escola de Química e Alimentos - Universidade Federal do Rio Grande (FURG) – Av. Itália, Km 08, CEP 96.201-900, Rio Grande- RS – Brazil
[4] Laboratório de Fungos de Importância Médica e Biotecnológica, Departamento de Biologia Molecular e Biotecnologia, Centro de Biotecnologia, UFRGS, Av. Bento Gonçalves, 9500, CEP 91501-970, Porto Alegre - RS – Brazil

Table S1. Water Contact Angle of PU films before and after UV irradiation in the presence of Oxygen or Acrylic Acid (AA) vapors).

UV irradiation time (min)	WCA (degrees)	
	O_2	AA
0	92	92
15	68	60
30	43	29
120	35	-

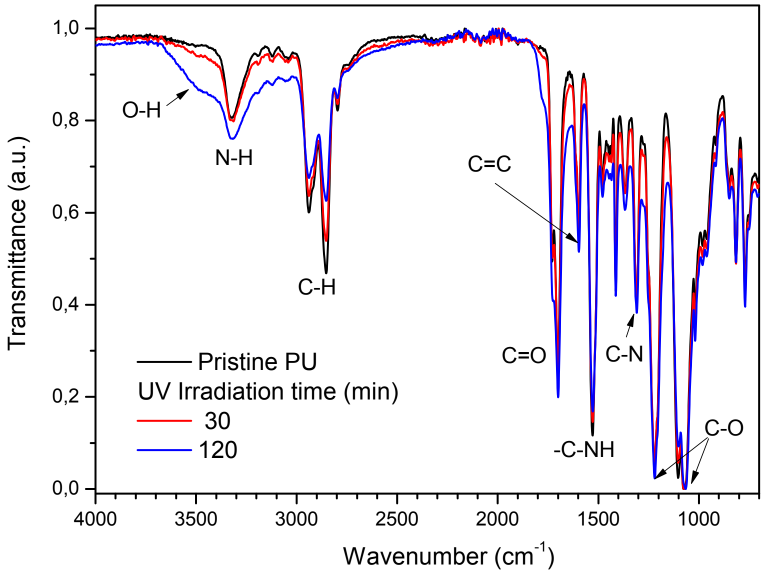

Figure S1. FTIR-ATR spectra of pristine PU films and treated ones with UV irradiation in the presence of oxygen for different times.

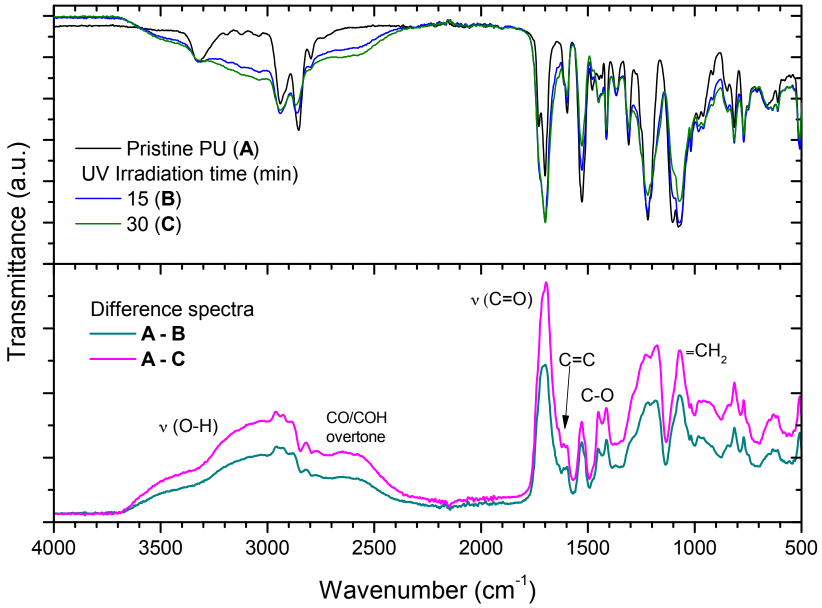

Figure S2. FTIR-ATR spectra of pristine PU films and treated ones with UV irradiation in the presence of AA vapors for different times (top) along with their difference spectra (bottom).

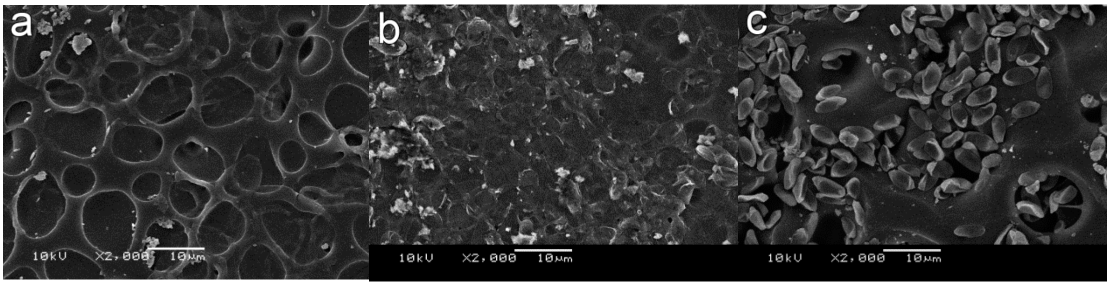

Figure S3. SEM images of pristine and UV treated PU films with Oxygen and incubated in the presence of entomopathogenic fungus *Metarhizium anisopliae* for 30 days. (a) without treatment; (b) UV treatment time of 30 min and (c) UV treatment time of 120 min.

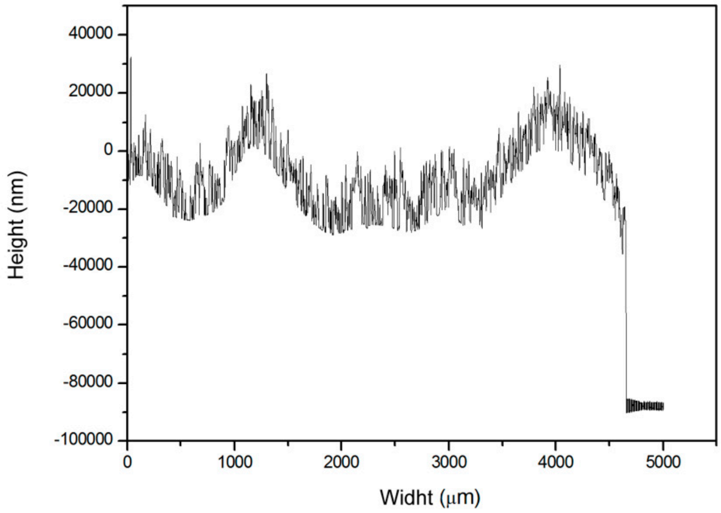

Figure S4. Profilometer cross-section of a PU film after UV irradiation in the presence of AA vapors for 30 min and incubated in the presence of the entomopathogenic fungus *Metarhizium anisopliae* for 30 days.

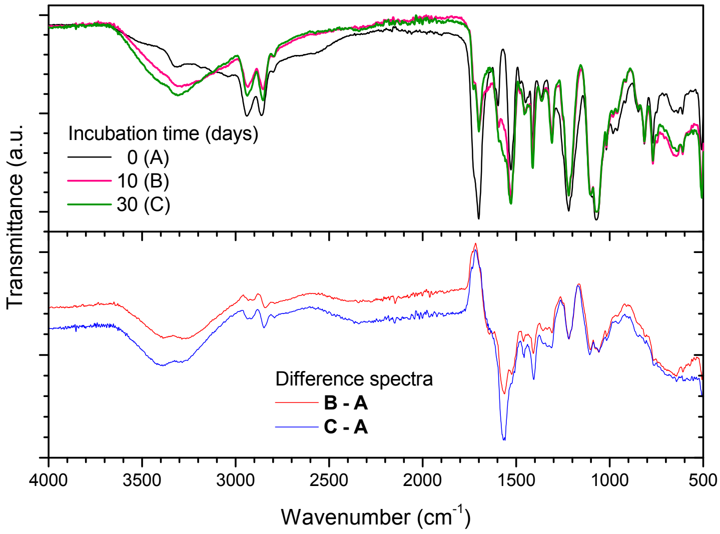

Figure S5. FTIR-ATR spectra of PU films treated with UV in the presence of AA vapors for 15 min without incubation and 10 and 30 days of incubation (top). The difference spectra are shown at the bottom. The spectra were slightly moved in the vertical direction for better presentation.
